# A Spectral Encoding Simulator for Broadband Active Illumination and Reconstruction-Based Spectral Measurement

**DOI:** 10.3390/s23104608

**Published:** 2023-05-10

**Authors:** Peng Jiang, Xiaoxu Wang, Zihui Zhang, Guochao Gu, Jifeng Li, Heng Wu, Limin He, Guanyu Lin

**Affiliations:** 1 Changchun Institute of Optics, Fine Mechanics and Physics, Chinese Academy of Sciences, Changchun 130033, China; 2 University of Chinese Academy of Sciences, Beijing 100049, China

**Keywords:** spectral measurement, spectral encoding, active illumination, compressed sensing

## Abstract

Spectral reflectance or transmittance measurements provide intrinsic information on the material of an object and are widely used in remote sensing, agriculture, diagnostic medicine, etc. Most reconstruction-based spectral reflectance or transmittance measurement methods based on broadband active illumination use narrow-band LEDs or lamps combined with specific filters as spectral encoding light sources. These light sources cannot achieve the designed spectral encoding with a high resolution and accuracy due to their low degree of freedom for adjustment, leading to inaccurate spectral measurements. To address this issue, we designed a spectral encoding simulator for active illumination. The simulator is composed of a prismatic spectral imaging system and a digital micromirror device. The spectral wavelengths and intensity are adjusted by switching the micromirrors. We used it to simulate spectral encodings according to the spectral distribution on micromirrors and solved the DMD patterns corresponding to the spectral encodings with a convex optimization algorithm. To verify the applicability of the simulator for spectral measurements based on active illumination, we used it to numerically simulate existing spectral encodings. We also numerically simulated a high-resolution Gaussian random measurement encoding for compressed sensing and measured the spectral reflectance of one vegetation type and two minerals through numerical simulations. We reconstructed the spectral transmittance of a calibrated filter through an experiment. The results show that the simulator can measure the spectral reflectance or transmittance with a high resolution and accuracy.

## 1. Introduction

Every object has its own unique spectral characteristics; as a result, the spectrum is regarded as the “fingerprint” of the object. The spectral reflectance or transmittance of an object contains considerable inherent physical information that can be applied in object classification [1,2], remote sensing [3,4,5,6], medical diagnosis [7,8,9], and image reproduction [10,11]. Spectral measurement methods based on active illumination address the constraints between the spectral resolution and signal-to-noise ratio of traditional spectral measurement methods and have gained attention due to their rapidity and accuracy [12]. Active illumination is an important component of computational optical imaging. This improves the imaging resolution and environmental adaptability by encoding space, time, polarization, or spectral information on the light source side. When the spectral signal is collected under broadband active illumination, the spectral reflectance or transmittance of the target object can be measured. In recent years, researchers have performed many studies on active-illumination-based spectral measurement techniques. Park et al. used a set of LEDs in a multiplexed sequence to illuminate a scene and applied a simple empirical linear model to measure the spectral reflectance [13]; however, although the measurement algorithm was accurate and fast, the combined spectrum of the LEDs was not continuously tunable due to the fixed spectrum of each LED. In actual use, this light source led to fitting difficulties with spectral encodings with a high resolution and accuracy. In addition to using LEDs as active illumination sources, Chi et al. proposed a new active illumination approach. They selected 16 channels using a set of 228 possible filters and used an optimized broadband filtered illumination to obtain multispectral reflectance information [14]. However, they used tungsten and xenon lamps with specific filters as light sources for active illumination. While this approach is simple, the spectral encoding of the nearly optimal illumination set, which was based on the designed Hadamard code, could not be fitted because the equivalent broadband filters were not physically available. The above traditional illumination methods are inflexible. Han et al. measured the reflectance of a scene using a digital light processing (DLP) projector as a light source with spectrally distinct illuminations and a high-speed camera [15]. DLP projectors equipped with RGB color wheels have only three fixed illumination spectra. Although high-speed spectrum switching can be achieved for dynamic scenes, accurate spectral measurements are difficult to attain. For a more accurate reconstruction of the spectral reflectance, Zhang et al. developed a deeply learned broadband encoding stochastic hyperspectral camera with an active measurement mode that used a broadband light source and random filter to illuminate the target object. With this approach, accurate and dynamic measurements of the spectra in the entire field of view were obtained by detecting the target spectral reflectance [16]; however, the random filter used in the directional design was difficult to manufacture.

To address the various deficiencies in the existing active illumination light sources, we designed a spectral encoding simulator (SES) based on spectral tuning technology. In reconstruction-based spectral measurements, there are various methods for spectral encoding. A commonly used method is performed at the receiver side, usually using devices such as filters for encoding. One of the other methods is performed at the light source side, encoding the spectrum of the initial light source into a specific spectrum (the premise is that active illumination is applied to spectral measurements), and our SES is an active encoding light source.

In recent years, an increasing number of scholars have applied digital micromirror devices (DMDs) in spectral tuning. These devices control the wavelength and intensity of the spectrum through two dimensions, namely, the DMD rows and columns. For example, in 2005, MacKinnon et al. proposed a spectral programmable engine (SPLE) that used a DMD to output a specific spectral distribution (SPD), which has been used in the fields of biochemistry and biomedicine [17]. This system was an early prototype of a programmable spectral light engine based on a DMD, which has inspired various spectral light engines that have been applied in different fields. In 2006, Chuang et al. proposed a DMD pattern-scanning calibration method and a digital programmable light spectrum synthesis system, which was applied to the synthesis of various infrared C-band (1530–1565 nm) spectral profiles [18]. In 2017, Luo et al. proposed a programmable light source in the visible range based on the combination of a prism and an echelle grating [19]. Rice et al. developed a visible hyperspectral image projector (HIP). This projector has a double-DMD symmetrical structure, with the DMDs used as the spectral engine and spatial engine. The HIP outperforms conventional digital light processing (DLP) projectors because it has hyperspectral image projection capabilities [20]. Hirai et al. proposed a projector with a programmable light source (Optronic Laboratories OL490) and a DMD. The projection principle was the same as that of DLP projectors, and the proposed projector achieved spectral image projection similar to the HIP [21]. In addition to the abovementioned techniques, spectral light engines have been developed in various fields, such as stellar simulation [22], lasers [23], and spectral calibration [24,25,26]. In addition, there are some commercially available spectral light engines, such as Optronic Laboratories OL490 and the recently developed Chromatiq Spectral Engine (CSE) from Energetic (Hamamatsu). These spectral light engines use gratings to achieve good and smooth spectral matching; however, they cannot achieve a broad spectral range or high brightness. While the abovementioned spectral light engines are all based on DMDs, there are some problems in using them for spectral measurements. Active illumination sources for spectral measurements require a broad spectral range, high spectral encoding accuracy, and high optical throughput. Thus, the light source is better suited for using a prismatic spectral imaging system.

The main work of this study is to present a method of applying the SES to active-illumination-based spectral measurement and to verify the feasibility of the method using numerical simulations. We designed the SES based on spectral tuning technology. The SES uses the flexible spatial light modulation capability of the DMD to simulate spectral encoding in the broadband range of 300–1100 nm, which provides a basis for broadband active illumination and the reconstruction of spectral measurements. The SES uses a prismatic spectral imaging system to image the spectral image onto the DMD. The spectral dimension of the image corresponds to the dimension of the DMD micromirror columns, and the spatial dimension corresponds to the dimension of the DMD micromirror rows. Therefore, the on–off state of the DMD micromirror columns determines the wavelength of the output spectrum, and the number of micromirrors in the on state in each column determines the intensity of this wavelength. After the hardware structure was determined, we verified the high optical throughput of the SES through numerical calculations and analysis. To accurately simulate spectral encoding, a spectral encoding simulation method based on a convex optimization algorithm is used to calculate the optimal state of each DMD micromirror, and the controller controls the on–off state of 1920 × 1080 micromirrors according to the calculated optimal state. Furthermore, we theoretically analyzed the spectral encoding simulation capability and performance of the SES. These are the preconditions for its application in the field of spectral measurement. To verify the applicability of the SES in spectral measurements, we used the SES to simulate some existing spectral encoding schemes. Moreover, we also used the SES to perform a Gaussian random spectral encoding, achieving compressed-sensing-based spectral measurements. The theoretical analysis and numerical simulation results show that the SES can be used to achieve accurate complex spectral encoding simulations, and the compressed-sensing-based spectral measurements using this light source show a priority in the spectral reconstruction accuracy, because the SES compensates for the low accuracy in the spectral encoding simulations near the high end of the wavelength tuning range. The proposed SES can be used not only for high accuracy active-illumination-based spectral measurements but also as a simulation and verification platform for various spectral encoding schemes because of its ability to achieve high-resolution and accurate spectral encoding simulations.

The main structure of the paper is as follows: Section 1 is the introduction. In Section 2, the principle (Section 2.1) and structure (Section 2.2) of the SES are introduced, and the optical throughput is numerically simulated (Section 2.3). In Section 3, the spectral encoding simulation principle (Section 3.1) is introduced, and, after the position–wavelength function is numerical simulated, the model of the spectral response function is established (Section 3.2). Then, the spectral resolution of the SES is numerically simulated; after that, the spectral encoding simulation method is finally introduced (Section 3.3). In Section 4, the spectral encoding simulation capability and performance is numerically simulated. In Section 5, the applicability of the SES in spectral measurement is verified by the numerical simulation of the spectral encoding designed by various groups (Section 5.1) and the numerical simulation of spectral measurement based on compressed sensing (Section 5.2). In Section 6, the performance of the SES is verified by spectral encoding simulation capability experiments (Section 6.1) and compressed sensing experiments (Section 6.2). Section 7 is the conclusion.

## 2. System Principle and Structures

### 2.1. System Principle

**Figure 1:**
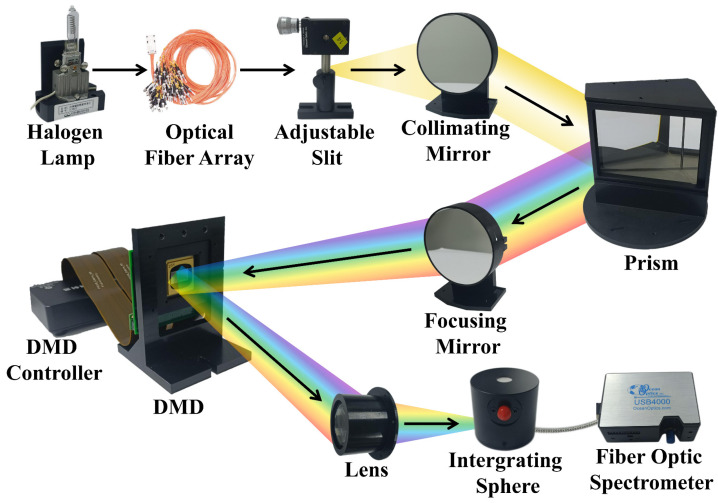
The hardware principle of the system.

The hardware principle of the simulated system is shown in Figure 1. The DMD controller controls the state of the 1920 × 1080 micromirrors according to the target SPD to encode the spectrum and then outputs the spectrum through the integrating sphere. The output of the integrating sphere is measured by the fiber optic spectrometer to evaluate the spectral encoding simulation accuracy.

### 2.2. System Structure

The main body of the SES uses the Czerny–Turner structure. The ray of the initial light sources (1000 W halogen lamp) is converged to the slit through a closely arranged fiber array and passes through an off-axis parabolic mirror (collimating mirror), a prism (dispersion element), and a spherical mirror (focusing mirror), imaging on the DMD. The spatial light modulation characteristics of the DMD are used to modulate the spectral image. The light is finally output through the converging lens and integrating sphere. The slit is 0.1 × 6 mm, the numerical aperture of the object is 0.12, the vertical magnification is 1.83, and the image height is 10.98 mm. In order to achieve a wide operating spectral range of 300–1100 nm and high optical throughput, the prismatic spectral imaging system and the fiber array are needed. The fiber array consists of 48 fibers with a diameter of 105 microns and a numerical aperture of 0.12. They are closely arranged in a 1 × 48 array to illuminate the slit. It is just enough to fill the entire 0.1 × 6 mm slit. The fibers on the other side are dispersed to couple more energy.

**Figure 2:**
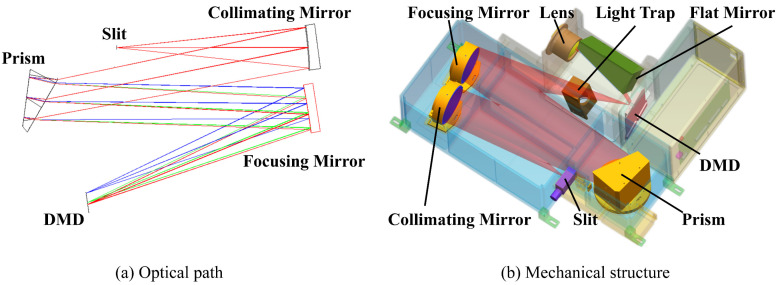
The optical–mechanical structure of the system: (**a**) the design of the optical path of the system; (**b**) the design of the mechanical structure.

The design of the optical path of the system is shown in Figure 2a, and the design software is zemax 14.2. Figure 2b shows the design of the mechanical structure, and the design software is ug 10.0.

### 2.3. Optical Throughput of the System

As an active illumination light source, the SES needs to have a high optical throughput to achieve spectral measurements with high signal-to-noise ratios. In order to improve the optical throughput of the SES and broaden the operating band, we used a prism made of JGS2 quartz. In addition, all reflective surfaces were simulated as coated with aluminum, and the front surface of the prism was coated with MgF2.

The use of the fiber array can also improve the optical throughput. The 48 optical fibers are equipped with 3 mm diameter grin lenses and placed at a distance of 50 mm from the 1000 W halogen lamp. In an ideal situation, the energy entering the fiber array is the product of the irradiance of the halogen lamp at 50 mm, the total area of all the grin lenses, and the coupling efficiency. For the same light source and coupling mode, the illuminance and coupling efficiency can be considered equal, so the optical energy gain is the ratio between the area of the fiber array and the area of the slit directly irradiated by the light source. This ratio is equal to approximately 300.

**Figure 3:**
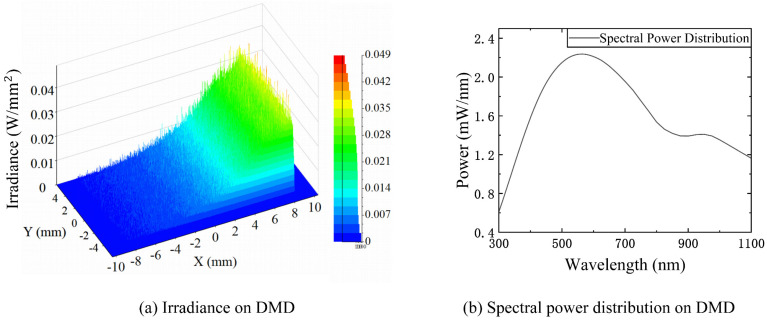
The optical throughput of the system: (**a**) the irradiance on DMD; (**b**) the spectral power distribution on DMD.

In the software simulation (the simulation software is lighttools 6.0), we set the light source spectrum as a 1000 W halogen lamp spectrum. The energy entering the slit was 1.94 W, and all the working surfaces were linked with the corresponding film characteristics. The simulation results are shown in Figure 3. Figure 3a shows the irradiance distribution on the DMD surface and Figure 3b shows the spectral power distribution on the DMD surface. The light energy utilization efficiency through the slit was 68.72%. However, the average diffraction efficiency with blazed or concave gratings was less than 50%, and they cannot be used with a broad spectral range. If other optical components are included, the light energy utilization rate decreases. For example, the initial light source of the CSE is directly used to illuminate the slit, and the CSE uses a concave grating, which operates in the range of 380–780 nm. The results of spectral measurements measured by the CSE have a low signal-to-noise ratio.

## 3. Spectral Encoding Simulation Method and Model

### 3.1. Spectral Encoding Simulation Principle

**Figure 4:**
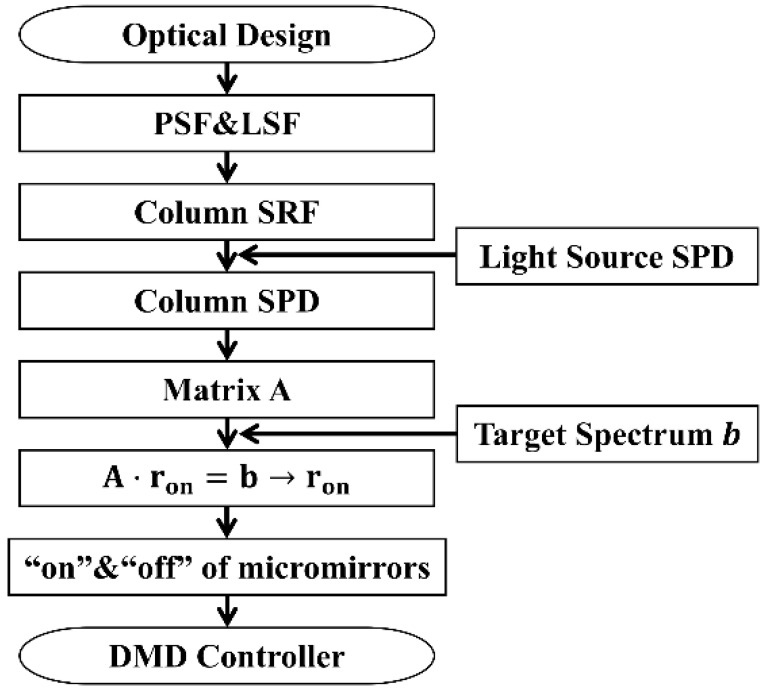
Flow chart of the spectral encoding simulation method. The PSF and LSF can be obtained by optical design, and the DMD column SPD can be calculated. The spectral matrix A is combined with all the SPDs. After the target spectrum is set, the state of DMD can be calculated. The state of DMD is fed into the controller.

The spectral encoding simulation principle, which is also the main process of the spectral measurement simulation, is shown in Figure 4.

**Figure 5:**
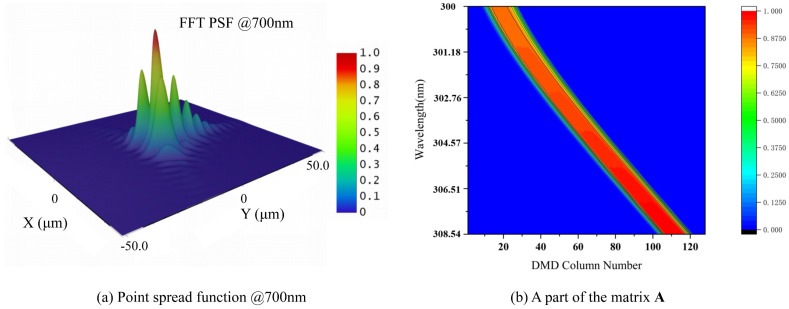
The spectral encoding simulation models: (**a**) the point spread function exported from optical design software; (**b**) contour map of a part of the matrix A.

As shown in the flow chart in Figure 4, based on the optical design, we can obtain the point spread function (PSF) and line spread function (LSF). The PSF describes the response of the imaging system to a point light source. Figure 5a shows the PSF in the on-axis field of view at 700 nm (the software is zemax 14.2). The LSF, which can be obtained by integrating the PSF in the slit length direction, describes the response of the imaging system to a line light source. The LSF can be used to obtain the spectral response function (SRF) of each DMD micromirror column. The product of the SRF and the initial light source SPD is the column SPD. All DMD column SPDs are divided into column vectors according to the central wavelength of the corresponding DMD micromirror column. These vectors are combined into a matrix known as spectral matrix A. Therefore, the row vector in matrix A represents the intensity of the corresponding wavelength in all DMD columns. The column vector in matrix A represents the SPD in the whole spectral band corresponding to the DMD column. Figure 5b shows a contour map of a part of matrix A. The horizontal axis represents DMD micromirror columns 1 to 128. The vertical axis represents wavelengths ranging from 300 nm to 308.54 nm. The blue parts in Figure 5b are all 0, which indicates that the micromirrors in this column do not receive light with the corresponding wavelength.

Spectral matrix A and a convex optimization algorithm can be used to determine the state of each DMD micromirror, leading to accurate spectral encoding simulations. The detailed encoding simulation steps are described in Section 3.2 and Section 3.3.

### 3.2. Model of Spectral Response Function

**Figure 6:**
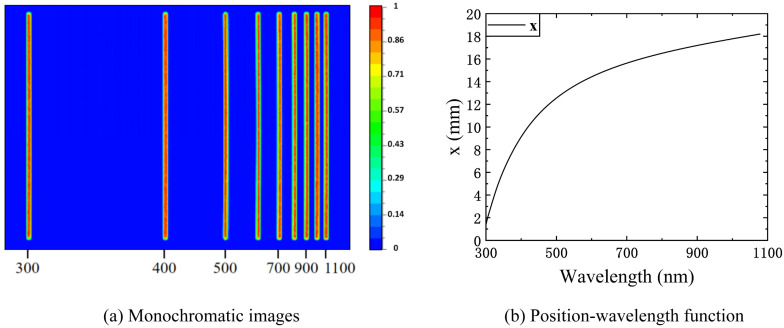
The positions of monochromatic images at different wavelengths on the image plane: (**a**) the distribution of monochromatic images at 9 wavelengths (the distance between 2 adjacent wavelengths is 100 nm) on the image plane; (**b**) the correlation of position to wavelength.

As shown in the flow chart in Figure 4, the SPD on each micromirror must be known to modulate the spectral image on the image plane by the DMD. The SPD on each micromirror is determined by the product of the initial light source SPD (discussed in Section 4.1) and the spectral response function (SRF) on that micromirror. The SRF is an inherent property of the prismatic spectral imaging system, which is mainly determined by the spectroscopic principle and the efficiency of the optical system. Spectrometers have inherent spectral smile effects. Through monochromatic ray tracing, we find that the spectral smile effect of the SES system is not significant, and the edge and center positions of the monochromatic image with the most severe smile effects are only three micromirrors apart. However, it will be a great challenge to find the SRFs of all micromirrors on the DMD, and the simulation accuracy is not significantly improved by calculating the SRFs of all micromirrors. Therefore, we assume that the micromirror SRFs in each DMD micromirror column are the same and that these SRFs are uniformly distributed in the spatial dimensional direction. Therefore, it is necessary to calculate only the sum of micromirror SRFs in each single DMD micromirror column. The SRF of the prismatic spectral imaging system is shown in Equation (1).

(1) $$SRF(\lambda )=rect\left(\frac{x\left(\lambda \right)}{b}\right)*\left[LSF\left(x\left(\lambda \right)\right)*rect\left(\frac{x\left(\lambda \right)}{a}\right)\right]\tau \left(\lambda \right),$$

where * represents the convolution operation, a is the slit width, b is the micromirror width, and Rect is a rectangular function. When the independent variable is between −0.5 and 0.5, it equals 1; otherwise, it equals 0. The former rectangular function represents the response function of the micromirror, and the latter represents the slit function. For each micromirror, its center is defined as zero, and x is the position in this micromirror coordinate system. The spectral power received by the micromirror can be regarded as the response of the micromirror to the translation and superposition of each monochromatic image of the incident slit, and this process is equivalent to the convolution of the micromirror response function and the response function of the incident slit. τ(λ) is the system optical efficiency, and $LSF(x\left(\lambda \right))$ is the LSF at the position corresponding to the DMD micromirror column, which can be obtained by integrating the PSF in the slit length direction after the optical design has been determined. x(λ) is the position coordinate along the dispersion direction on the image plane. There is a mapping relationship with the wavelength in the spectral dimension. Using the method of monochromatic ray tracing, the monochromatic image position coordinates can be simulated (the simulation software is lighttools 6.0). As shown in Figure 6a, the distribution of the monochromatic images from left to right is 300 nm, 400 nm, 500 nm, 600 nm, 700 nm, 800 nm, 900 nm, 1000 nm, and 1100 nm on the image plane. Figure 6b shows the correlation of x to wavelength.

**Figure 7:**
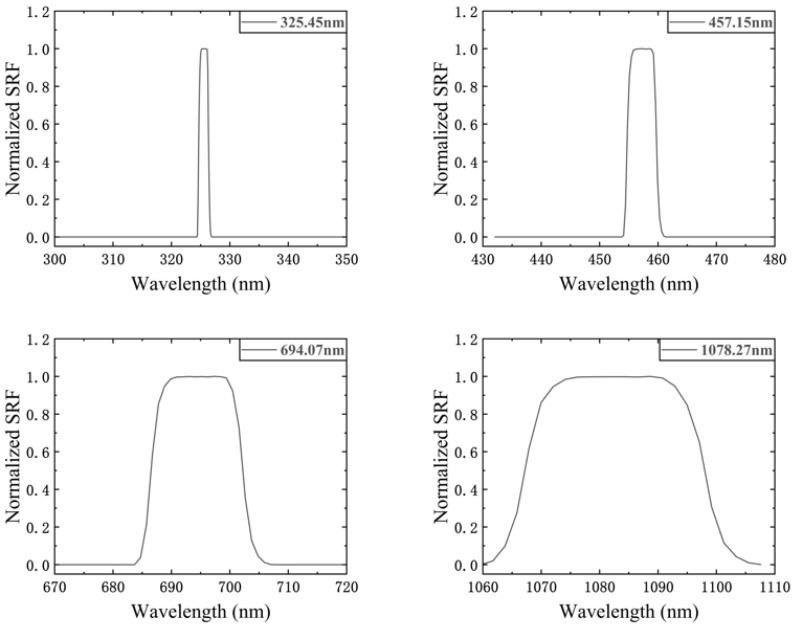
The normalized SRF curves of the four DMD columns with center wavelengths of 325.45 nm, 457.15 nm, 694.07 nm, and 1078.27 nm.

When the position–wavelength mapping relationship is applied in the SRF equation, the SRF curve can be obtained as shown in Figure 7. A total of 1575 DMD micromirror columns are covered in the range of 300–1100 nm, so a total of 1575 SRF curves are needed. The normalized SRF curves of the four DMD micromirror columns with center wavelengths of 325.45 nm, 457.15 nm, 694.07 nm, and 1078.27 nm are shown in Figure 7.

**Figure 8:**
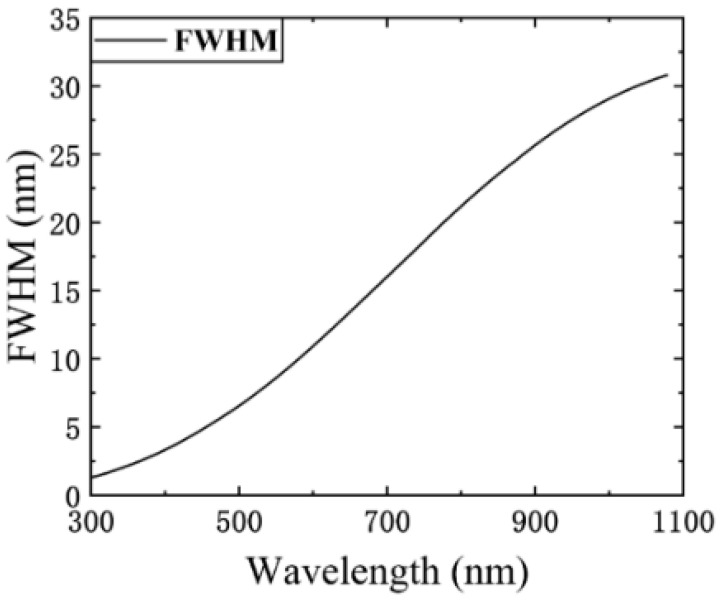
The spectral resolution of SES (numerical simulation).

The full width at half maximum (FWHM) of the SRF also represents the spectral resolution, as seen in Figure 7 and Figure 8. The FWHM varies at different locations and increases with increases in the wavelength. This places a constraint on the spectral encoding simulation performance of the SES. The specific constraint relationship is discussed in Section 4.2.

### 3.3. Spectral Encoding Simulation Method

**Figure 9:**
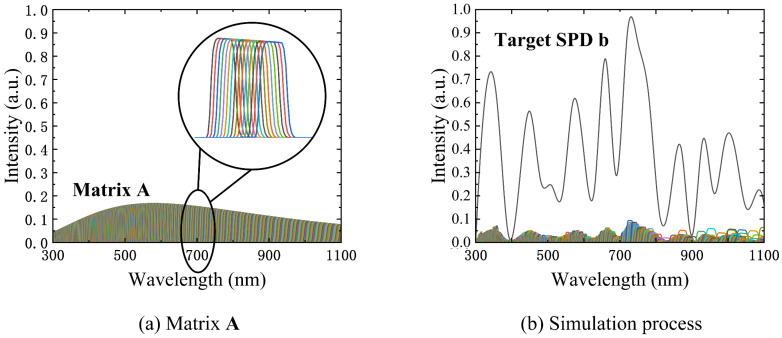
The spectral encoding simulation process: (**a**) the 1575 × 1575 SPD matrix A (expanded by column); (**b**) the simulation process of a random target SPD (the sum of all the modulated columns is close to the target SPD b).

The SRFs of the 1575 DMD micromirror columns are discussed in Section 3.2. These SRFs are multiplied by the initial source SPD to obtain the SPDs of the 1575 DMD micromirror columns. Each SRF is discretized into a 1575-dimensional column vector according to the central wavelength of the corresponding DMD micromirror column and then combined into a 1575 × 1575 matrix known as SPD matrix A. The spectral encoding simulation process is shown in Figure 9. Figure 9a shows matrix A, which consists of the SPDs of the 1575 DMD micromirror columns. A halogen lamp with a 5000 K color temperature is used as the initial light source. Figure 9b shows the simulation process of a random target SPD. The simulation process can be modeled as follows:

(2) $$A\cdot r_{on}=b,$$

where $r_{on}$ and b are 1575-dimensional column vectors. $r_{on}$ is the unknown to be determined and represents the proportion of the micromirrors in the “on” state in each DMD micromirror column, which has a value between 0 and 1. A value of 0 means that the micromirrors in the column are all off, while a value of 1 means that the micromirrors in the column are all on. b is obtained by dividing the target SPD by the spectral reflectance of the micromirrors, the transmittance of the lens and the reflectance of the integrating sphere diffuse reflector.

To solve for $r_{on}$, the 2-norm is used as the evaluation function to calculate $r_{on}$, and the result of $r_{on}$ is used to guide the switching of each DMD micromirror column. The equation then becomes a convex optimization problem with 1575 constraints, as shown below:

(3) $$\hat{r_{on}}=\arg\underset{r_{on}}{\min}\left({\left\|A\cdot r_{on}-b\right\|}_{2}\right),\;s.t.\;\;0\leq r_{on}\leq 1.$$

We use a convex optimization algorithm to solve this equation [27,28], which can calculate the proportion of the micromirrors that should be in the on state in each column with minimum deviation. As a toolkit, cvx is simple to use. By entering the code according to Equation (3) and importing A and b, the result can be easily obtained. $r_{on}$ is multiplied by the number of DMD micromirror rows and rounded to the nearest integer to determine the number of micromirrors that should be in the on state in each column. To minimize the error caused by the spectral smile mentioned in Section 3.2, the code is designed so that each column of DMD micromirrors turns on from the middle to both ends.

## 4. Spectral Encoding Simulation Capability and Performance

Matrix A and the target SPD b in the algorithm described in the previous section affect the spectral encoding simulation capability and spectral encoding simulation performance of the SES, respectively. These effects are discussed in the following sections. (All of the following programs were written in python).

### 4.1. Spectral Encoding Simulation Capability

**Figure 10:**
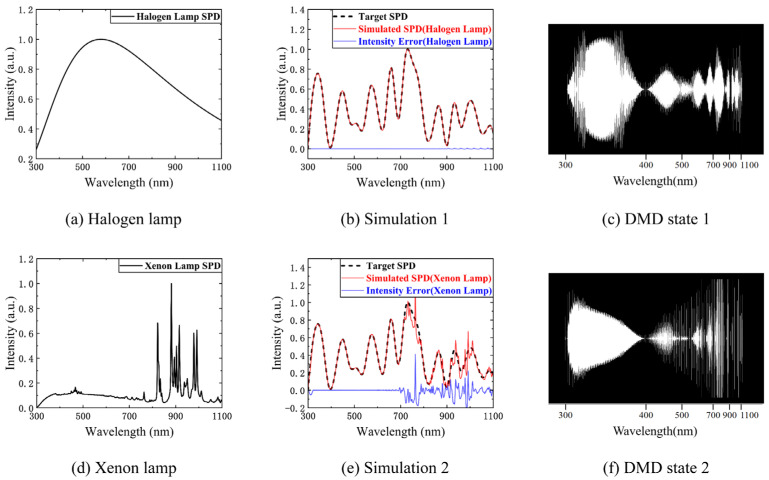
The spectral encoding simulation capability: (**a**) the halogen lamp SPD; (**b**) the numerical simulation result using the halogen lamp; (**c**) the DMD state when the halogen lamp is used for simulation (black pixels represent the off state and white pixels represent the on state); (**d**) the xenon lamp SPD; (**e**) the numerical simulation result using the xenon lamp; (**f**) the DMD state when the xenon lamp is used for simulation.

Matrix A determines the spectral encoding simulation capability, and the product of the SPD of the initial light source and the SRFs of the SES determines matrix A. For different types of initial light sources, the spectral encoding simulation capability is different. A halogen lamp (with a color temperature of 5000 K) and a xenon lamp were used for simulations. The SPDs of these two initial light sources are shown in Figure 10a,d, and a random target SPD was simulated with two initial light sources. The numerical simulation results are shown in Figure 10b,e. The simulation capability of the SES with xenon lamps with characteristic spectra was inferior to that of the SES with halogen lamps in the range of the characteristic spectral bands. Therefore, to improve the simulation capability of the SES, the initial light source should be halogen lamps without characteristic spectra. Figure 10c,f show the DMD state when the halogen lamp and xenon lamp are used for simulations, respectively (black pixels represent the off state and white pixels represent the on state).

### 4.2. Spectral Encoding Simulation Performance

The target SPD b determines the spectral encoding simulation performance. Due to the nonlinear spectroscopic characteristics of the prism, the spectral resolution (FWHM of the SRF) decreases with increases in the wavelength. Figure 8 shows the trend of the change in the FWHM of the SRF with the wavelength. The maximum FWHM is 30 nm in the range of 300–1100 nm. Therefore, the resolution of the target SPD b should be greater than 30 nm to obtain an accurate spectral encoding simulation. Clearly, the resolution of b can increase with a decrease in the range of wavelengths.

**Figure 11:**
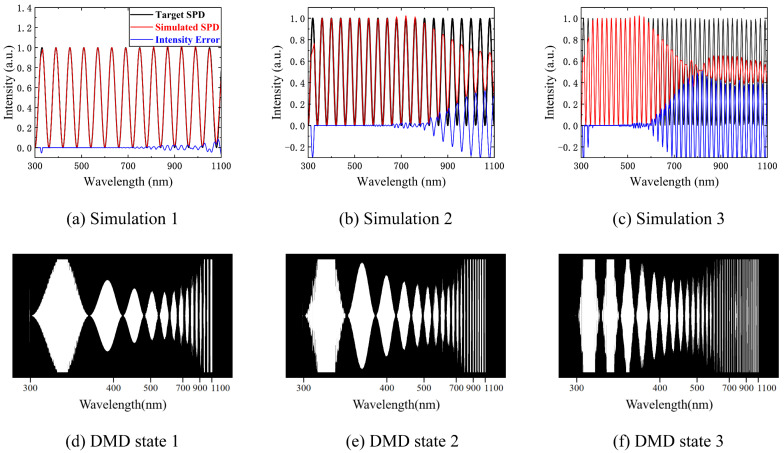
The spectral encoding simulation performance: (**a**) the numerical simulation result of the extreme case of 30 nm resolution; (**b**) the numerical simulation result of the extreme case of 20 nm resolution; (**c**) the numerical simulation result of the extreme case of 10 nm resolution; (**d**) the DMD state of the 30 nm resolution case; (**e**) the DMD state of the 20 nm resolution case; (**f**) the DMD state of the 10 nm resolution case.

Figure 11a–c show extreme cases of 30 nm, 20 nm, and 10 nm resolutions, respectively. Figure 11d–f show the DMD states of the three numerical simulation cases. The root mean square errors (RMSEs) of the three cases are 1.22%, 8.05%, and 13.83%, respectively. Since our numerical simulations are in extreme states, the determined RMSE corresponds to the maximum deviation of the three cases.

The above numerical simulation results show that the error increases with increases in the wavelength, and the main reason is that the SES uses a prismatic spectral imaging system. The broad bandwidth near the high end of the wavelength tuning range could limit the simulation capability and performance.

## 5. Applicability of the SES in Spectral Measurement

### 5.1. Simulation of Spectral Encoding

The SES can be used to simulate spectral encoding schemes in existing spectral measurement methods based on active illumination. We numerically simulated spectral encoding schemes designed by various groups.

**Figure 12:**
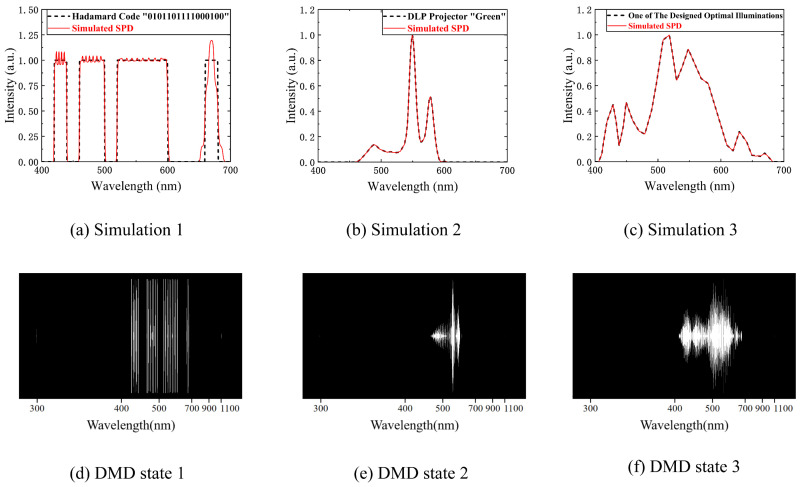
The numerical simulation of spectral encoding designed by various groups: (**a**) the numerical simulation result of spectral encoding based on Hadamard code; (**b**) the numerical simulation result of the green spectral illumination channel in the RGB of the DLP projector; (**c**) the numerical simulation result of one of the optimal illumination SPDs; (**d**) the DMD state when simulating spectral encoding based on Hadamard code; (**e**) the DMD state when simulating the green spectral illumination channel; (**f**) the DMD state when simulating one of the optimal illumination SPDs.

Chi et al. designed the spectral encoding of a nearly optimal illumination set based on the Hadamard code. Since physically perfect narrow bandpass filters do not exist, they used the SPD information of a real narrow bandpass interference filter (Edmund Optics) to simulate nearly optimal illumination [14]. Our SES simulated spectral encoding based on the Hadamard code numerically, which cannot be achieved with light sources and filters, and the numerical simulation results are shown in Figure 12a.

Han et al. used a DLP projector as an illumination source and combined it with a high-speed camera to measure spectral reflectance [15]. This approach has the advantage of being able to obtain spectral reflectance measurements in dynamic scenes by using the high-speed switching illumination SPD of the DLP projector, whose core component is a DMD. Our SES can achieve the same SPD switching speed as the DLP projector. When combined with a high-speed camera, it can also obtain the spectral reflectance measurements of dynamic scenes. Figure 12b shows the performance of the SES in numerically simulating the green spectral illumination channel in the RGB of the DLP projector.

Fu et al. presented a simple and efficient convolutional neural network (CNN)-based spectral reflectance recovery method with optimal illumination. They designed an illumination optimization layer to optimally multiplex illumination spectra in a given dataset or to design the optimal one under physical restrictions [29]. We numerically simulated one of the designed optimal illumination SPDs for spectral encoding. The numerical simulation results are shown in Figure 12c.

In summary, our SES achieves good numerical simulation for the illumination spectral encoding used by existing active illumination methods.

Compared to the spectral measurements achieved by Zhang et al. using a light source and random filter combined with their deeply learned broadband encoding stochastic hyperspectral camera [16], we used the SES to replace the light source and random filter and simulated the measurement of spectral reflectance or transmittance by the compressed sensing method numerically, as discussed in the Section 5.2.

### 5.2. Spectral Measurement Based on Compressed Sensing

Compressed sensing (CS) is a sampling theory that obtains measurements under a specific measurement basis **Φ** and represents the signal to be sampled sparsely in the time domain by means of a sparse basis **Ψ**. The sparse signal can be finally recovered by various algorithms. CS can reduce the number of samples while maintaining signal integrity, which can be applied to the measurement of spectral reflectance or transmittance. The principle of CS is shown in the following equation:

(4) y=ΦΨS,

where y is the $N_{1}$ × 1 observation vector formed by M measurements ($N_{1}=M$), **Φ** is the $N_{1}$ × $N_{2}$ observation matrix, **Ψ** is the sparse matrix of $N_{2}$ × $N_{2}$, and **S** is the sparse signal of $N_{2}$ × 1. According to compressive sensing theory, the key to measuring whether **S** can be compressively losslessly sampled is the restricted isometry property (RIP). The independent and identically distributed Gaussian random measurement matrix can be the universal compressive sensing measurement basis. The sensing matrix formed by the Gaussian random measurement matrix and most sparse bases can satisfy the RIP; therefore, we choose the Gaussian random matrix as the measurement basis. In addition, we use the overcomplete dictionary as the sparse basis and construct the sparse basis with a Gaussian function as the parent function, which can be well sparsed for the spectral reflectance or transmittance data.

As shown in Equation (5) in the Gaussian function, the central wavelength is μ, and the FWHM is $\sqrt{8ln2}$. This is equal to the spectral sampling interval $\lambda_{s}$. After stretching the Gaussian function $2^{m}$ times (m is an integer), we can obtain a new Gaussian function with a stretched FWHM. N times (n is an integer) the stretched FWHM is the center wavelength offset to yield the new Gaussian function that covers the entire spectral range. The set of functions forms a sparse basis **Ψ**, as shown in Equation (6).

(5) $$g\left(\lambda ;\mu ,\sigma \right)=\frac{1}{\sqrt{2\pi }\cdot \sigma }\exp\left[-\frac{{\left(\lambda -\mu \right)}^{2}}{2\sigma^{2}}\right],$$

(6) $$\left\{\begin{array}{l} \Psi_{m,n}\left(\lambda \right)=\sqrt{2^{m}}\cdot g\left(\lambda ;2^{m}n\lambda_{s},\frac{2^{m}\lambda_{s}}{\sqrt{8\ln2}}\right);\\ 1\leq 2^{m}\leq \frac{\lambda_{\max}-\lambda_{\min}}{\lambda_{s}}\;;\;\frac{\lambda_{\min}}{\lambda_{s}2^{m}}\leq n\leq \frac{\lambda_{\max}}{\lambda_{s}2^{m}}\;;\;m,n\in Z \end{array}\right\}.$$

Finally, the convex optimization algorithm is used to measure the spectral reflectance or transmittance. The algorithm is shown in Equation (7).

(7) $$\hat{S}=\arg\underset{S}{\min}\left({\left\|\Phi \Psi S-y\right\|}_{1}+0.05{\left\|S\right\|}_{1}\right).$$

**Figure 13:**
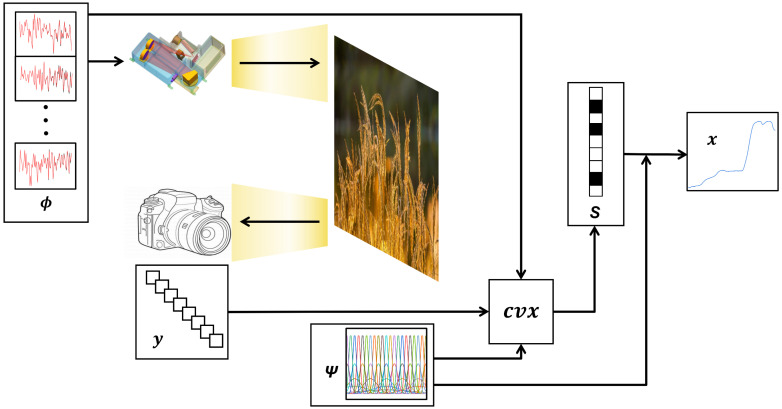
The process of spectral reflectance measurement. *Φ* is the measurement basis, which is simulated by SES, y is the signal obtained by the detector, and *Ψ* is the sparse basis. The above three are used as input quantities, and the sparse signal *S* is obtained by the *cvx*. x is the measured spectral reflectance or transmittance signal obtained by multiplying *Ψ* and *S*.

After the measurement basis **Φ**, the sparse basis **Ψ**, and the algorithm are determined, the spectral measurement process is proposed, as shown in Figure 13. The $N_{1}$ row vectors in **Φ** are regarded as the spectral encoding. The SES simulates this spectral encoding as the active illumination SPD, and the camera is used to acquire M reflectance or transmittance signals y. The sparse spectral reflectance or transmittance signal **S** is measured by the convex optimization algorithm (cvx) [27,28], and **ΨS** is the measured spectral reflectance or transmittance signal.

For the illumination, the integrating sphere can be replaced with a fiber coupler to improve energy utilization. In addition, light guides, which have larger diameters than optical fibers and higher energy utilization than optical fibers and integrating spheres, can also be used as illumination elements for the SES. The commercially available quartz light guides and liquid light guides can directly couple the light from the SES. The use of a fiber coupler or light guides for illumination can meet spectral measurement requirements.

**Figure 14:**
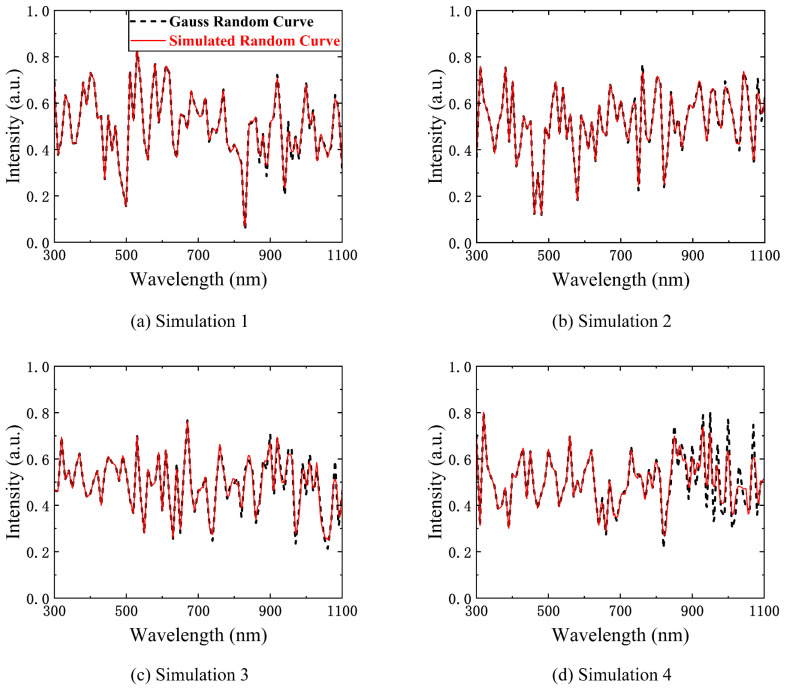
Four of the numerical simulations of Gauss random encoding. The black curves are Gauss random curves and the red curves are simulated random curves. The simulation performance is shown in the four numerical simulations.

The most important and difficult part of compressed sensing is the physical implementation of the measurement basis **Φ**. Our SES can easily implement a Gaussian random encoding because of its flexibility. We choose a spectral sampling interval of 10 nm ($N_{2}$= 80) to generate a Gaussian random matrix. This is regarded as the spectral encoding. The numerical simulation results are shown in Figure 14. Four of the numerical simulations are shown, with the bottom right figure showing the worst numerical simulation results.

**Figure 15:**
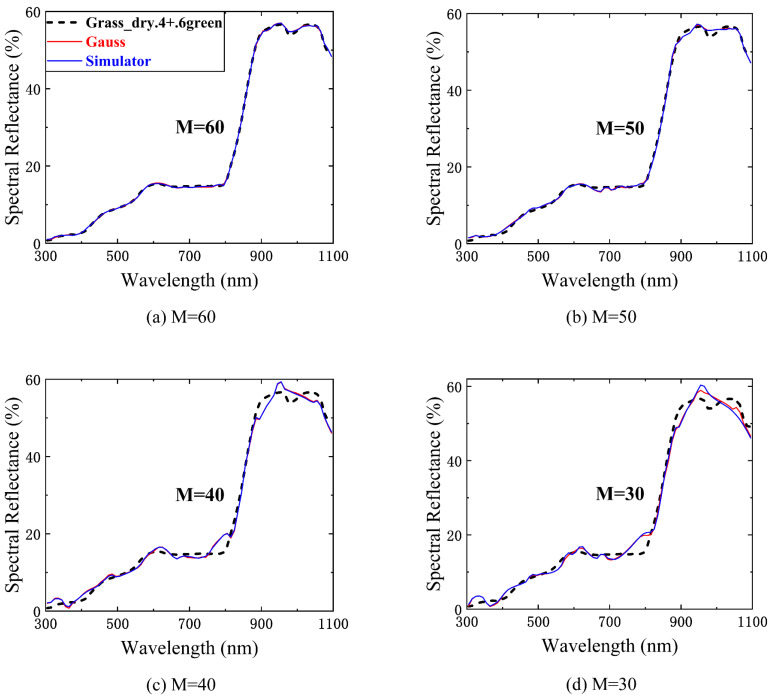
The numerical simulation of Grass_dry.4+.6green spectral reflectance with the real Gaussian random matrix and the SES simulated matrix as the measurement encoding (M is 60, 50, 40, and 30).

According to the RIP, all column vectors in the Gaussian random matrix should be approximately orthogonal, that is, with weak correlation. It is calculated that the mean of the absolute value of the cross-correlation coefficient of the generated Gaussian random matrix is 0.087 and the maximum value is 0.428. The mean value of the absolute value of the cross-correlation coefficient of the simulated matrix is 0.0901 and the maximum value is 0.434, which can meet the requirement of weak correlation. Figure 15 shows the numerical simulation of Grass_dry.4+.6green from the US Geological Survey (USGS) spectral reflectance database [30] with the real Gaussian random matrix and the SES simulated matrix as the measurement encoding. The values for the number of measurements M are 60, 50, 40, and 30, and the product of the measurement encoding and the spectral reflectance is regarded as the measurement value y.

The numerical simulation results show that the reconstruction ability of the two measurement bases gradually diverges (the deviation between the blue line and the red line in the figure) as M decreases. The following table (Table 1) lists the RMSEs of the spectral measurements obtained with the two measurement matrices using different numbers of measurements. The simulated matrix and the real Gaussian random matrix have basically the same performance on the spectral measurement, and there is no apparent superiority or inferiority relationship between them. This indicates that the simulation error on the Gaussian random matrix does not affect the measurement accuracy of spectral reflectance or transmittance.

**Figure 16:**
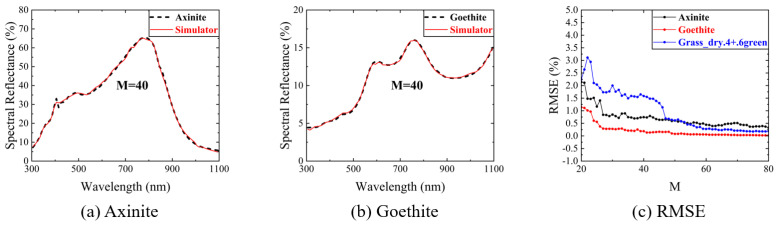
The spectral reflectance measurement of two minerals at a sampling rate of 50% (M = 40): (**a**) the spectral reflectance measurement of Axinite_HS342.3B; (**b**) the spectral reflectance measurement of Goethite_WS222; (**c**) the change in RMSE for three spectral reflectance measurements with measurement time M.

In the USGS database, we randomly selected the spectral reflectance of two minerals (Axinite_HS342.3B & Goethite_WS222) [30] and measured them at a sampling rate of 50% (M = 40). The results are shown in Figure 16a,b. Figure 16c shows the change in the RMSE for three spectral reflectance measurements with measurement time M. The measurement deviation has a significant downward trend as the measurement time increases and tends to stabilize after reaching 58% (M = 47). The RMSE is less than 1%.

In summary, with the flexible spectral encoding simulation capability, the SES can simulate a variety of complex spectral encoding, such as Gaussian random measurement encoding, spectral encoding based on deep learning, and Hadamard-based spectral encoding, so as to achieve accurate spectral reconstruction based on active illumination.

## 6. Validation Experiments

To verify the performance of the SES, we designed two validation experiments to verify its spectral encoding simulation capability and applicability of compressed sensing, respectively.

### 6.1. Experiments to Verify the Capability of Spectral Encoding Simulation

**Figure 17:**
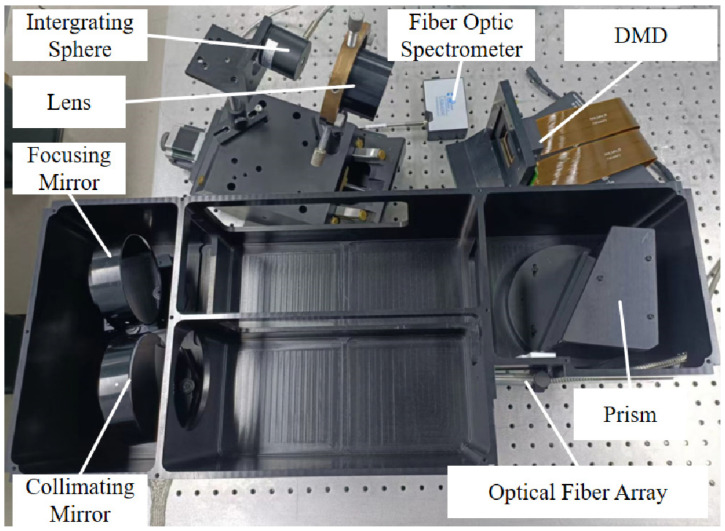
The drawing of the experimental platform.

To verify the spectral encoding simulation capability and performance of SES, we mounted the experimental platform as shown in Figure 17.

The initial light source is a 1000 W spectral irradiance standard lamp 210,701 (National Institute of Metrology, Beijing, China). The optical fiber array is RH-48-127-8-L (Ruihe, Beijing, China). The prism, the collimating mirror, the focusing mirror, and the lens were specialized manufactured by the Changchun Institute of Optics, Fine Mechanics and Physics, China. The DMD is the Discovery F4110 (Jinhua Fldiscovery, Jinhua, China), and the core DMD chip is the DLP9500 (Texas Instruments, Dallas, TX, USA). The DMD control program is F4110DMDControlerV2.0, which is provided by Jinhua Fldiscovery. The integrating sphere is the JFQ-25 (Hangxin, Guangzhou, China). The fiber optic spectrometer is the USB4000 (Ocean Optics, Orlando, FL, USA). All other control and data acquisition software were written in Python 3.6.

The USB4000 (Ocean Optics) has a high response at 450–850 nm, so our experiments were operated at 450–850 nm.

**Figure 18:**
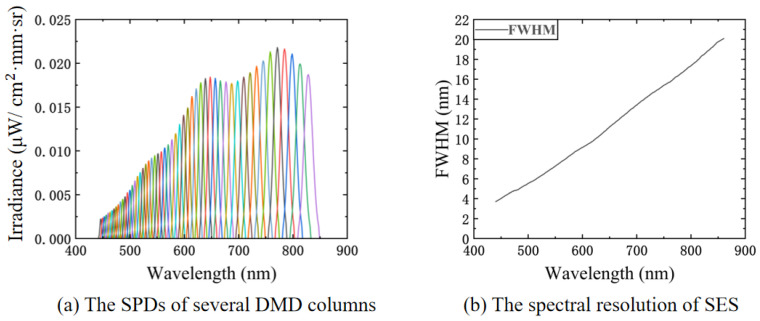
The tests of basic SES performance: (**a**) the DMD column SPDs of SES; (**b**) the spectral resolution of SES.

After the experimental platform was mounted, we tested the main performance of the SES, including the SPDs received by each micromirror column and the spectral resolution of the system. We tested the SPDs of the micromirror columns by opening the single column. Figure 18a shows the result of several SPDs. The tested SPDs were also used to calibrate the SES. Based on the SPDs, we solved the spectral resolution of the SES as shown in Figure 18b.

**Figure 19:**
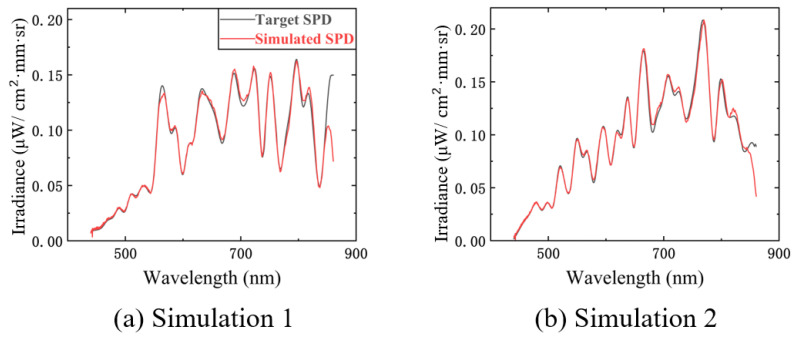
The experimental simulations of two random spectral encodings: (**a**) the experimental simulation result (red simulated SPD in the figure) of spectral encoding 1 (black target SPD in the figure); (**b**) the experimental simulation result of spectral encoding 2.

We experimentally simulated random spectra with a 10 nm resolution at 450–850 nm after the experimental platform was mounted and calibrated, and the results are shown in Figure 19a,b. The average relative errors of the two experiments were 3.07 and 3.81%. The RMSE values were 0.0065 $\mu W/cm^{2}\cdot mm\cdot sr$ and 0.0043 $\mu W/cm^{2}\cdot mm\cdot sr$. The main reason for the errors was that the spectral and radiometric calibrations of the experimental platform were not precise enough.

### 6.2. Experiments to Verify the Applicability of Compressed Sensing

**Figure 20:**
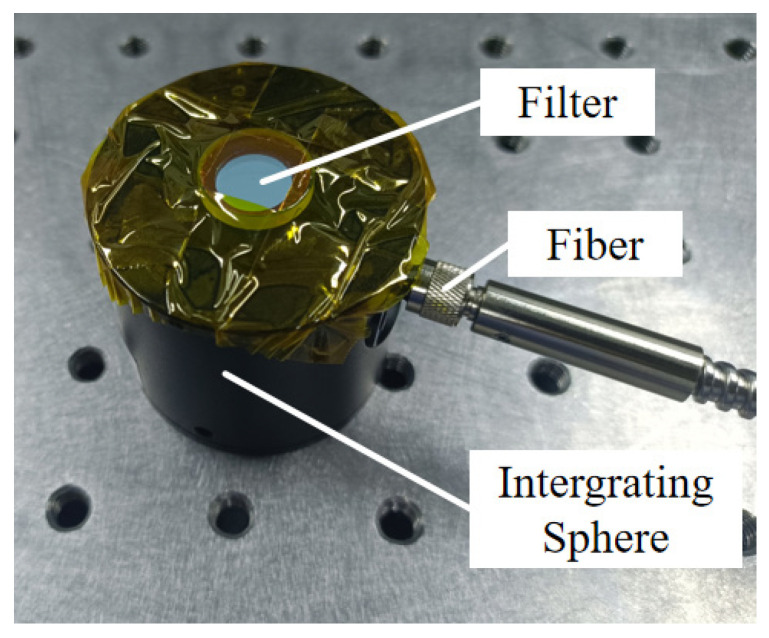
The drawing of the experimental sample.

To verify the applicability of compressed sensing, we used an existing calibrated filter as an experimental sample and pasted the filter in front of the integrating sphere, as shown in Figure 20.

**Figure 21:**
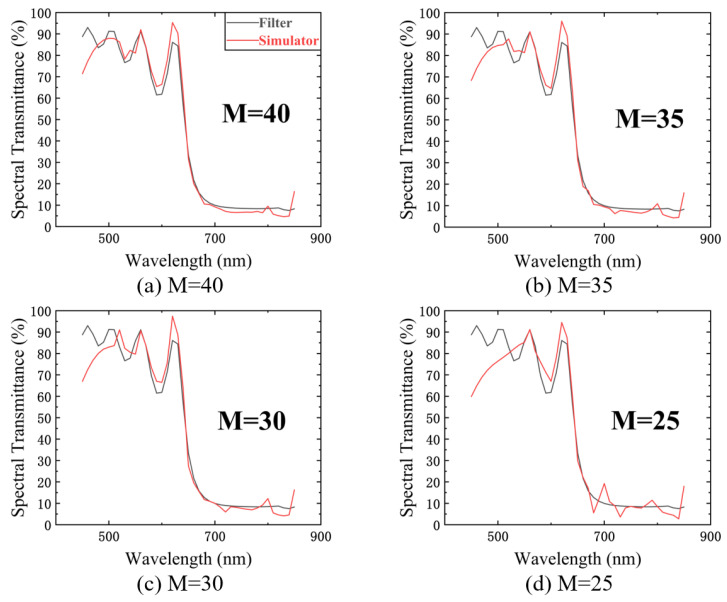
The transmittance measurements with different measurement time M (M = 40, 35, 30, and 25). The black curves in the figures are the calibrated transmittance of the filter. The red curves are the measurement results obtained from transmittance reconstruction experiments.

We used M’ random spectra (one random spectrum corresponds to one measurement, so the M’ is equal to the measurement time M) to illuminate the filter with a sampling resolution of 10 nm, which is the same as the sampling resolution of the filter calibration result, and then used a single-point detector to collect the energy signal. We finally used the compressed sensing algorithm to reconstruct the filter spectral transmittance. The results are shown in Figure 21a–d, which show the reconstruction effect for M values of 40, 35, 30, and 25, respectively. Since compressed sensing requires known random spectra, we still used the USB4000 to measure the random spectra, so we reconstructed the spectral transmittance at 450–850 nm.

**Figure 22:**
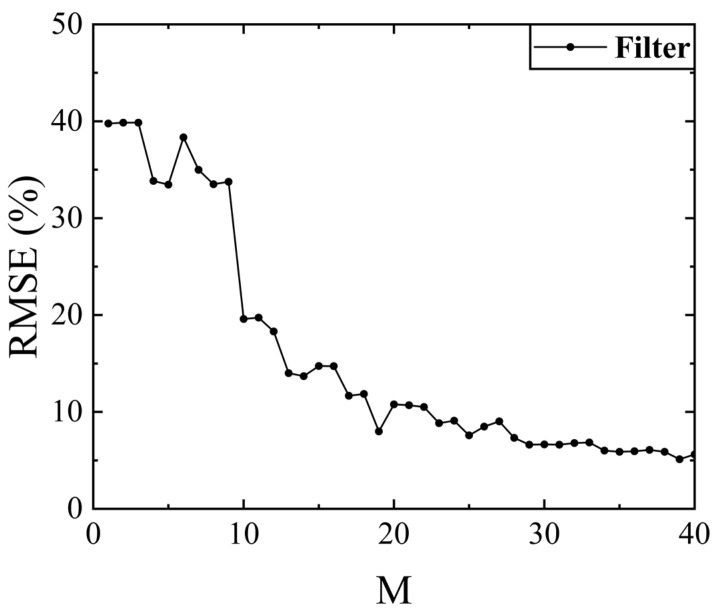
The RMSE for the spectral transmittance measurements with measurement time M.

The causes of the reconstruction errors in the figure are the detector measurement errors, especially the time drift error and the detector response error. The former leads to a gradual increase of the error when the number of measurements increases, and the latter leads to a poorer reconstruction in the low response band (450–550 nm). Figure 22 shows the change in the RMSE for the spectral transmittance measurements with measurement time M.

## 7. Conclusions

Since the light sources used in existing methods of spectral measurement cannot effectively implement the designed active illumination spectral encoding, we designed a spectral encoding simulator for the broadband of 300–1100 nm and calculated the spectral response function on the image plane of the prismatic spectral imaging system and the spectral distribution on the DMD. According to the spectral distribution on the DMD, we used a convex optimization algorithm to design a spectral encoding simulation method. In addition, we explored the impact of different initial light sources on the spectral encoding simulation capability and the impact of different target spectral resolutions on the spectral encoding simulation performance. The simulation data show that the use of tungsten halogen lamps without characteristic spectra can improve the spectral encoding simulation capability. The SES can simulate spectral encoding at 30 nm, 20 nm, and 10 nm resolutions in the extreme case with RMSEs of less than 1.22%, 8.05%, and 13.83%, respectively. We also simulated the spectral encoding for most of the existing active illumination methods. The results show that the SES can achieve the spectral encoding designed by other research groups. Finally, based on compressed sensing theory, we simulated a 10 nm resolution Gaussian random measurement encoding and measured the spectral reflectance or transmittance. The simulation results show that the goodness of fit of the spectral measurement is higher than 0.998 when the sampling rate is larger than 50%. The recovery rate (success rate) of the system is high because the cvx we use is stable, and the accuracy is acceptable.

Depending on the spectral modulation characteristics of the DMD, the main advantage of our SES is that it can flexibly simulate various spectral encodings and combine various algorithms to measure spectral reflectance or transmittance for active illumination. The main disadvantage of our SES is that the optical throughput of the initial light source is reduced due to the use of the slit. To compensate for this defect, we use a prismatic spectral imaging system. We are exploring an optical design without slits to improve the optical throughput, and this problem is expected to be resolved in future work. Our SES also has other potential applications, such as in star simulators [22], calibration light sources [24,25,26], and other scenes requiring tunable spectra. The SES can even be used as a simulation and verification platform for various forms of spectral encoding schemes and spectral reconstruction verification.

## Figures and Tables

**Table 1:** The RMSEs of spectral measurement with two measurement matrices.

M	RMSE of Gauss (%)	RMSE of SES (%)
80	0.181	0.185
70	0.232	0.211
60	0.29	0.275
50	0.599	0.619
40	1.636	1.584
30	1.773	2

## Data Availability

Data underlying the results presented in this paper are available in Refs. [27,28,30].
